# Optimization of Quercetin‐Assisted Silver Nanoparticles Synthesis and Evaluation of Their Hemocompatibility, Antioxidant, Anti‐Inflammatory, and Antibacterial effects

**DOI:** 10.1002/gch2.202100075

**Published:** 2021-10-20

**Authors:** Azam Chahardoli, Pouria Hajmomeni, Mahnaz Ghowsi, Farshad Qalekhani, Yalda Shokoohinia, Ali Fattahi

**Affiliations:** ^1^ Department of Biology Faculty of Science Razi University Kermanshah 6714414971 Iran; ^2^ Pharmaceutical Sciences Research Center Health Institute Kermanshah University of Medical Sciences Kermanshah 6734667149 Iran; ^3^ Ric Scalzo Institute for Botanical Research Southwest College of Naturopathic Medicine Tempe AZ 85282 USA; ^4^ Medical Biology Research Center Health Technologies Institute Kermanshah University of Medical Sciences Kermanshah 6734667149 Iran

**Keywords:** anti‐inflammatory activity, antimicrobial effect, coagulation time, hemolysis, QE‐AgNPs, quercetin

## Abstract

In the present study, different effective parameters (temperature, reaction time, and pH) on the synthesis of quercetin‐assisted silver nanoparticles (QE‐AgNPs) are optimized. These biogenic NPs are characterized by different physico‐chemical analyses, including transmission electron microscopy, X‐ray diffraction, Fourier transform infrared (FTIR) spectroscopy, and UV‐visible spectroscopy. In addition, the biological properties of QE‐AgNPs are evaluated through antioxidant, antimicrobial, anti‐inflammatory, hemolysis, and coagulation time assays. The formation of QE‐AgNPs is affected by different parameters. The optimum condition for the synthesis of QE‐AgNPs is attained at 70 °C and pH 7. Prepared QE‐AgNPs show a spherical shape with a crystalline nature and an average particle size of 20 ± 3.6 nm. The role of QE as a reducing and capping agent in the preparation process of QE‐AgNPs is demonstrated using FTIR analysis. These NPs with excellent antioxidant activity (82.3% at a concentration of 400 µg mL^−1^) and anti‐inflammatory properties (82.5% and 100% at concentrations of 37.25 and 500 µg mL^−1^, respectively), show good antimicrobial effects, particularly against *Staphylococcus aureus*. Furthermore, the results of the hemolytic and coagulation assay of QE‐AgNPs indicate their hemo‐compatibility. Therefore, hemo/bio‐compatible QE‐AgNPs with excellent and unique properties can be employed in different medicinal and pharmacological applications.

## Graphical abstract

In the current study, the optimal conditions for the synthesis of quercetin‐assisted silver nanoparticles (QE‐AgNPs) and their biological properties are investigated. Spherical QE‐AgNPs (20 ± 3.6 nm) with excellent anti‐inflammatory properties, blood compatibility, and antioxidant activity are effective against bacterial strains, particularly *Staphylococcus aureus*. Therefore, these NPs with unique properties can be a suitable option for various medicinal applications.

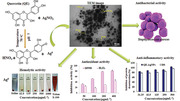

## 1 Introduction

Recently, the use of flavonoids in the field of phyto‐nanotechnology has attracted much attention because of the efficacy of these compounds in decreased risks of various chronic diseases and contributing to human health. Quercetin, a naturally occurring flavonoid, has biological activities such as antioxidant, antiviral, anti‐inflammatory, anticancer, antiallergic, and cardiovascular protectivity. Quercetin is a free radical scavenger,^[^ ^1^ ^]^ and platelet aggregation inhibitor, which could protect the heart, liver, and kidney.^[^ ^2^ ^]^ The presence of several hydroxyl (–OH) groups and the carbonyl fraction in the structure of flavonoids such as quercetin can lead to the chelation of metal ions. However, quercetin application is restricted owing to its low bioavailability, week solubility, and instability in a physiological medium, which limits its clinical and nutraceutical benefits.^[^ ^3^ ^]^ Thus, nanotechnology may be an alternative to address these limits and can be a promising procedure to build new formulations to improve the biological activity of quercetin and potentiate the activity of this molecule.^[^ ^4^ ^]^

There are limited reports about the synthesis of metallic nanoparticles (NPs) by reducing the pure flavonoid. For example, quercetin and other isolated flavonoids such as hesperidin, diosmin, naringin, and quercetin di/penta‐phosphate have been applied in the biosynthesis of silver nanoparticles (AgNPs) with a wide range of sizes, shapes, compositions, and physicochemical properties and improved bioactivities.^[^ ^5^, ^6^, ^7^, ^8^, ^9^, ^10^ ^]^ Antibacterial, antioxidant, and cytotoxic properties of the NPs synthesized using these compounds are noteworthy. Antibacterial activity of green synthesized AgNPs by flavonoids, quercetin, and lignin have been investigated against different bacterial strains.^[^ ^3^, ^5^, ^6^, ^7^, ^8^, ^10^ ^]^ however, according to our knowledge, there is no or very few studies on optimizing variables for efficient synthesis of quercetin‐assisted (QE)‐AgNPs and evaluation of various biological effects, particularly blood compatibility or anti‐inflammatory activity of these NPs.

Hence, the present study was designed to optimize AgNPs synthesis using quercetin as a reducing agent; QE‐AgNPs were analyzed by X‐ray diffraction (XRD), transmission electron microscopy (TEM), Fourier‐transform infrared spectroscopy (FTIR), and UV‐vis spectrophotometer. Furthermore, the biocompatibility of AgNPs was assessed to determine their antioxidant, antimicrobial, and anti‐inflammatory properties. In addition, their blood compatibility was tested with hemolysis and coagulation assay.

## 2 Results and Discussion

### 2.1 Synthesis and Characterization of QE‐AgNPs

**Scheme 1:**
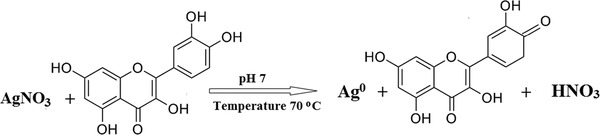
Synthesis mechanism of AgNPs using quercetin.

**Figure 1:**
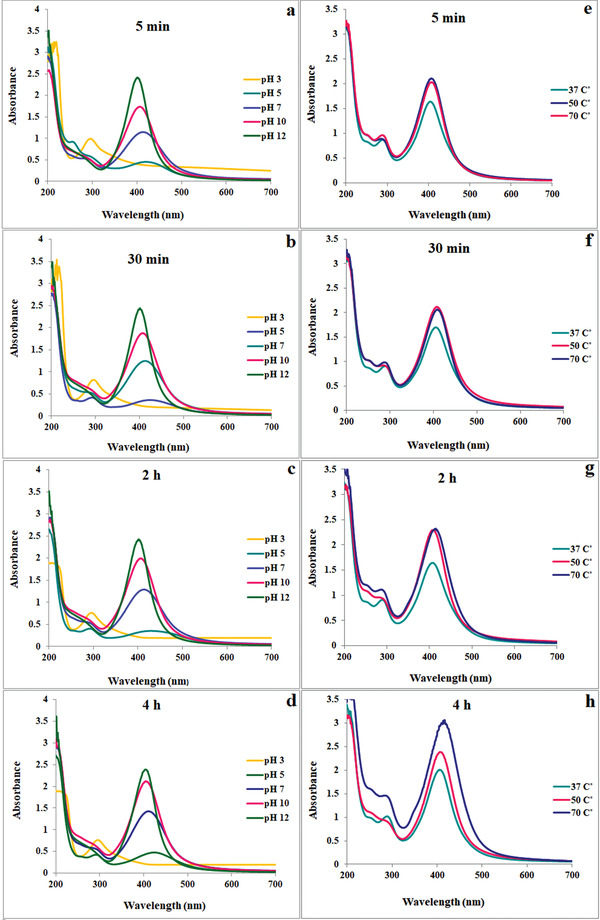
UV‐vis spectra of QE‐AgNPs at a–d) different pH of 3, 5, 7, 10, and 12 at room temperature and e–h) temperatures of 37, 50, 70 °C in pH of reaction solution at different time intervals of a,e) 5 min, b,f) 30 min, c,g) 2 h, and d,h) 4 h.

QE‐AgNPs were biosynthesized using quercetin (QE) as a reducing agent at different pH and temperatures, according to the reactions shown in **Scheme** **1**; the structure of quercetin contains a system of conjugated double bonds with five hydroxyl groups, which lead to a high reductive capacity.^[^ ^11^ ^]^ The reduction of Ag ions to form NPs was monitored by color changes and UV‐vis spectrophotometry and controlling the absorption spectra at regular time intervals. One of the crucial parameters affecting size, shape, and morphology of biogenic QE‐AgNPs is pH, which can be changed by the electrical charges of QE in reaction media and thus can change the reducing and capping capacity of QE, which effects the quick growth of NPs.^[^ ^8^ ^]^ In the present study, we evaluated pH 3, 5, 7, 10, and 12 at different time intervals (5 min, 30 min, 2 h, and 4 h) on the synthesis of QE‐AgNPs, which has been shown in **Figure** **1**a–d. There was no color changes observed in the reaction mixtures at low pH (pH 3) and thus the characteristic absorption peaks of the AgNPs at around 400–450 nm were not observed. It was detected that with a rise in pH, the absorption peak shifted from higher to lower wavelength of 431 (pH 5) to 413 nm (pH 7) and 404 nm (pH 10), within 1–5 min of reaction (Figure 1a). After 2 h of reaction (Figure 1c), it is shifted from 428 nm (pH 5) to 413 nm (pH 7) and 408 nm (pH 10), indicating an increase in the absorption intensity and a decrease in the size of prepared QE‐AgNPs. After 4 h of reaction time (Figure 1d), the maximum absorption was 424 nm at pH 5 and shifted to 411 nm at pH 7 and 406 nm at pH 10, which was close to reaction time 2 h and did not show significant alteration. Generally, the intensity of absorption increases with increasing pH and a rise in intensity suggests an enhancement in the AgNPs formation with time.^[^ ^12^ ^]^ At pH 12, higher intensity was observed and the maximum absorption was 404 nm after 2 and 4 h of reaction time. Therefore, the obtained results confirm that basic pH is more suitable for the synthesis of QE‐AgNPs.

**Figure 2:**
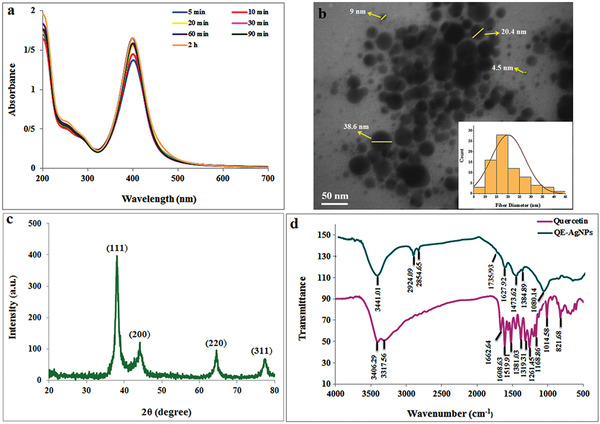
a) UV‐vis spectra, b) TEM image, c) XRD, and d) FTIR spectra of QE‐AgNPs at optimum condition.

Another parameter affecting the synthesis of QE‐AgNPs is the reaction temperature, which is the higher temperature, the reaction rate increased and the size of NPs decreased. Figure 1e–h presents the difference in the absorption spectra of biogenic QE‐AgNPs at tested temperatures of 37, 50, and 70 °C without changing the pH of the reaction solution (pH 4.1) in the various time intervals of 5 min, 30 min, 2 h, and 4 h. After 1 and 2 h of reaction time (Figure 1g), the absorption peak shifted from 408 nm at temperatures 37 and 50 °C to 406 nm at 70 °C and absorption intensity was increased by increasing of temperature resulting in increasing the reaction rate. After 4 h of reaction time (Figure 1h), it was detected that with a rise in temperature of reaction solution, the absorption peak shifted toward a higher wavelength (from 403 at 37 and 50 °C to 411 nm at 70 °C), which indicated that with longer reaction times, the silver ions and quercetin molecules get consumed faster and the growth of NPs inters to the secondary phase, which is particle aggregation. The particle size of QE‐AgNPs based on dynamic light scattering (DLS) analysis has been compared under different conditions in **Table** **1**. Based on these results, the optimum condition for the synthesis of QE‐AgNPs with small size was obtained after 2 h reaction at pH 7 and 70 °C (**Figure** **2**a). The UV‐vis spectra of QE‐AgNPs clearly show the strong surface plasmon resonance (SPR) band at 401 nm in optimum condition. A rise in absorbance was detected over time, demonstrating a development in the formation of QE‐AgNPs. Further analyses were performed using QE‐AgNPs synthesized at optimum condition.

**Table 1:** Size of synthesized QE‐AgNPs based on DLS at different pH and temperature values

Parameters	pH				Temperature [°C]		
	5	7	10	12	37	50	70
Size (nm)	261	99.8	112	263.2	207	104	85
Pdi	0.29	0.32	0.21	0.311	0.36	0.27	0.35

As seen in Figure 2b, TEM images under optimum conditions confirmed the spherical nature of QE‐AgNPs with an average size of 20 ± 3.6 nm. The crystal structure of QE‐AgNPs was determined using XRD. According to this analysis, the four distinct diffraction peaks at 38, 44.1, 64.5, and 77.3 indicate the crystalline nature of QE‐AgNPs, which corresponds to the value of the lattice plane indexed at (111), (200), (220), and (311) planes of the face‐centered cubic structure of QE‐AgNPs (Figure 2c). The average particle size calculated using the equation of the Debye–Scherrer from the XRD pattern was 24.4 nm, which is in line with TEM analysis. Chemical composition of reactants is involved in the synthesis of QE‐AgNPs analyzed by FTIR spectroscopy. As shown in Figure 2d, the broadband was observed at 3441 cm^−1^, which is assigned to O—H stretching vibration and can be associated with hydroxyl groups in the QE structure. The peak at 1736 cm^−1^ is related to the C=O stretch of carbonyls, carboxylic acids, aldehydes, esters, and saturated aliphatic. The peak at 1628 cm^−1^ is attributed to the N—H bend of amines and aromatic C=C in the carboxyl group. Also, a band at 1474 indicates the presence of C—C stretch of aromatics and a band at 1080 assigned to C—N stretch of amines and C—O stretching of primary alcohol. These results confirmed the reducing and stabilizing activity of quercetin in the synthesis of QE‐AgNPs.

### 2.2 Antioxidant Potential of QE‐AgNPs

**Figure 3:**
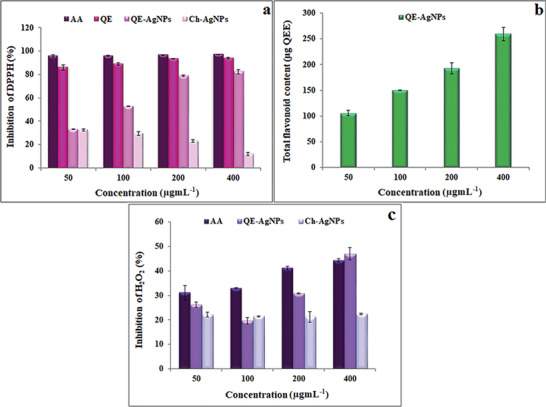
a) DPPH radical scavenging activity, b) quercetin content, and c) H_2_O_2_ scavenging activity of QE‐AgNPs at different concentrations of 50, 100, 200, and 400 µg mL^−1^.

The antioxidant properties of biologically synthesized NPs using flavonoid and phenolic compounds are related to the redox potential of these compounds, which allow them to perform as hydrogen donors, singlet oxygen scavengers, and strong reducing agents.^[^ ^13^ ^]^ In the current work, the antioxidant potential of QE‐AgNPs and QE compared to ascorbic acid (AA), as a standard antioxidant compound, and chemically synthesized AgNPs (Ch‐AgNPs) was evaluated by the inhibition of the diphenyl‐2‐ picrylhydrazyl (DPPH) free radical. As shown in **Figure** **3**a, there was an increase in scavenging DPPH radical by QE‐AgNPs in a dose‐dependent manner. While, DPPH scavenge activity of Ch‐AgNPs decreased in a dose‐dependent manner, which may be due to their small size, lack of suitable capping agent on their surface, and the highest potential of these chemically synthesized NPs in the generation of radical species. However, QE acted as a strong antioxidant in scavenging of DPPH radical even at low concentrations similar to standard compound (AA), which its inhibition activity estimated 86% at a concentration of 50 µg mL^−1^ and 94% at a concentration of 400 µg mL^−1^ (Figure 3a). The strong antioxidant activity of QE can be related to its chemical structure with more hydroxyl moieties, particularly in the B‐ring, which was previously reported.^[^ ^2^, ^14^ ^]^ QE‐AgNPs showed an inhibition percentage of 33.2% and 82.3% at the lowest and highest concentrations, respectively. The antioxidant activity of these NPs at higher concentrations was close to that of the AA standard antioxidant compound. The good antioxidant activity of QE‐AgNPs can be due to the existence of QE as a reducing and capping agent during the process of synthesis that was shown by evaluating the total flavonoid content based on QE content. As shown in Figure 3b, QE content in the structure of QE‐AgNPs increased in a dose‐dependent manner, and its content was 259.37 µg at the highest concentration of 400 µg mL^−1^. Also, the obtained results showed that the QE‐AgNPs at the highest concentration of 400 µg mL^−1^ had 47% H_2_O_2_ inhibitory activity (Figure 3c), which was higher than the AA standard compound with 44.3% H_2_O_2_ inhibitory potential at the same concentration. As described above, these properties of QE‐AgNPs could be due to the presence of QE on the surface of biosynthesized particles. Compared with QE‐AgNPs and AA standard compound, Ch‐AgNPs did not show significant H_2_O_2_ inhibitory activity at different concentrations.

Thus, QE‐AgNPs with high antioxidant activity can be used as a strong nano‐antioxidant in the pharmaceutical and medicinal industry. As we know, the toxicity, health concerns, multiple unpredictable, and deleterious effects of chemically synthesized AgNPs to prokaryotic, eukaryotic cells and multicellular organisms and the environment have been reported and mostly attributed to the generation of reactive oxygen species (ROS) and creating oxidative stress.^[^ ^15^, ^16^ ^]^ These synthetic NPs can cause adverse effects indirectly on primary or secondary organs (such as the cardiovascular or central nervous systems) and lead to hepatotoxicity, neurotoxicity, inflammatory, pulmonary inflammation, genotoxicity, and cytotoxicity.^[^ ^16^, ^17^ ^]^ However, surface coating of biogenic AgNPs by nontoxic organic compounds can be a reason for their safety in the biomedical and healthcare fields.^[^ ^18^ ^]^ The biogenic compounds with antioxidant effects can keep cells and tissues from the destructive effects of ROS produced during inflammation or other diseases.^[^ ^19^ ^]^ Therefore, biogenic QE‐AgNPs coated with antioxidant quercetin can be an effective nanomaterial for medical purposes compared to chemically synthesized AgNPs with oxidative activity.

### 2.3 Antimicrobial Effects of QE‐AgNPs

The antimicrobial effects of QE‐AgNPs were assessed against different gram‐negative and gram‐positive bacterial strains and also, a fungus strain of *Candida albicans* using standard methods of minimal inhibitory concentration (MIC) and minimum bactericidal concentration (MBC). Based on the obtained results in **Table** **2**, the strong antibacterial action of QE‐AgNPs was observed at 200 µg mL^−1^ against *S. aureus*, while it was 400 µg mL^−1^ for other bacterial strains. The MBC of QE‐AgNPs was same as MIC for all pathogens. This promising antibacterial activity of QE‐AgNPs against *S. aureus* can be used in the food industry and medicinal applications by helping to protect the skin and wound. It can be applied in hospital equipment because this opportunistic pathogen is the main cause of hospital infections. Along with bacteria, *C. albicans*, as a fungus opportunistic pathogen, can also cause several fatal diseases nosocomial infection with a mortality rate of about 40%.^[^ ^20^ ^]^ As shown in Table 2, QE‐AgNPs indicated MIC and MBC values of 400 µg mL^−1^ against *C. albicans*. In addition, in the present study, the aqueous solution of QE was also tested, which its antimicrobial effect with MIC and MBC values calculated at a concentration of 1 mg mL^−1^. These results were in accordance with studies of Marrez et al. on the effects of quercetin against *S. aureus*, *E. coli*, and *P. aeruginosa*.^[^ ^21^ ^]^ Therefore, QE‐AgNPs can be an effective antimicrobial candidate for application in different medicinal fields, e.g., in some products as wound dressings, catheters, stents, blood bags, and biomaterial implants.

**Table 2:** Antimicrobial activity of QE‐AgNPs

Microorganisms	QE‐AgNPs	
	MBC [µg mL^−1^]	MIC [µg mL^−1^]
*Pseudomonas aeruginosa*	400	400
*Staphylococcus aureus*	200	200
*Escherichia coli*	400	400
*Candida albicans*	400	400

Although some possible mechanisms for antimicrobial activity of AgNPs have been offered, but the exact mechanism has not yet been clarified. Nevertheless, possible antimicrobial mechanisms of QE‐AgNPs can be attributed to the function of intact AgNPs or Ag^+^ cations released from these NPs. The Ag^+^ cations bind to the negatively charged bacterial cell wall and sulfhydryl groups in enzymes and proteins leading to denaturation of protein, inhibition of respiratory chain enzymes, prevention of cell division, increased cellular oxidative stress, and ultimately cell death.^[^ ^22^, ^23^ ^]^ Also, the intake of AgNPs by directly attaching to the cell wall surface and penetrating through it and connecting to proteins in bacterial membranes or by increased ROS generation can lead to membrane damage, leakage of cellular contents, interaction with intracellular structures and biomolecules (proteins, DNA, ribosomes, and enzymes) in cytoplasm, and death as a consequence.^[^ ^24^ ^]^

### 2.4 Hemolytic Activity of QE‐AgNPs

**Figure 4:**
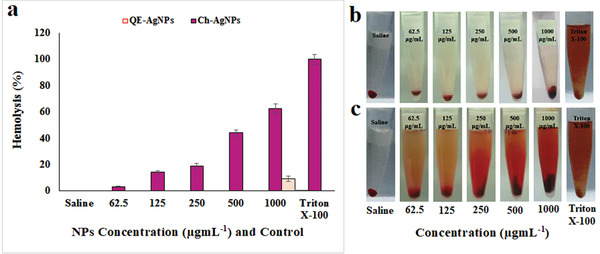
The hemolytic activity of biogenic Ag‐NPs at various concentrations (62.5–1000 µg mL^−1^). a) Percentage of hemolysis induced by QE‐Ag‐NPs and Ch‐AgNPs. Photographs of RBCs after exposed to b) QE‐Ag‐NPs and c) Ch‐AgNPs. Triton‐X‐100 and saline were, respectively, used as positive control and negative control.

The blood compatibility of AgNPs has critical role in their biomedical applications because blood is a primary target against toxic effects of these NPs. When the AgNPs entering the circulatory system, it leads to rupturing of RBCs and the release of hemoglobin, which may cause anemia, jaundice, other pathological diseases, and kidney failure. Hence, coating or surface modification of AgNPs is necessary to improve their stability and decrease their toxicity.^[^ ^25^ ^]^ Therefore, in the current work, the hemolytic potential of QE‐AgNPs was carefully assessed to determine their blood compatibility. As is shown in **Figure** **4**a,b, our results showed QE‐AgNPs suspension had 9.29% hemolysis at 1000 µg mL^−1^, and the other suspension of QE‐AgNPs (62.5, 125, 250, and 500 µg mL^−1^) had no hemolytic effects. On the other hand, Ch‐AgNPs (chemically synthesized AgNPs with diameter of 8 nm) at 1000, 500, 250, 125, and 62.5 µg mL^−1^ caused 62.5%, 44.11%, 18.98%, 14.01%, and 3.31% hemolysis, respectively, that were significantly different from saline (a negative control) (Figure 4a,c). The hemolytic action levels for all concentrations of QE‐AgNPs were less than the hemolytic activity levels of Ch‐AgNPs significantly (at *p* < 0.05; the significance of results is not shown in Figure 4). The hemolytic activity is a cytotoxicity model that was evaluated in the present study. The QE‐AgNPs did not show hemolytic activity in any concentrations up to 500 µg mL^−1^, but a concentration of 1000 µg mL^−1^ caused significant hemolysis in comparison to the negative control. Also, all the concentrations of QE‐AgNPs solutions had less hemolytic effects in contrast to Ch‐AgNPs solutions. In the previous reports, e.g., Vanaraj et al. synthesized AgNPs using methanolic quercetin with 35–80 nm indicated 4.58% hemolysis at concentration 120 µg mL^−1^,^[^ ^6^ ^]^ while in our results QE‐AgNPs with an average size of 20 ± 3.6 nm did not show any hemolysis charge at concentrations below 1000 µg mL^−1^, which shows our better results. One of the reasons for this different effect is probably the exposure time of RBCs against AgNPs, so that in our study, RBCs were exposed to QE‐AgNPs for 1 h, while in the abovementioned study, the incubation time was 4 h, which caused higher toxicity. In addition, the hemolysis effects of NPs attributed to their physico‐chemical properties such as size, charges, surface chemistry, and localized‐SPR effect.^[^ ^22^ ^]^ For example, it is reported that AgNPs with a smaller sizes than 50 nm cause higher hemolysis due to their higher uptake by erythrocytes.^[^ ^22^ ^]^ Therefore, Ch‐AgNPs with a small size of 8 nm caused higher hemolysis compared to QE‐AgNPs. The surface chemistry of NPs is another factor that plays a major role in RBCs toxicity, thus the presence of biomolecules in the surface of NPs leads to less toxic effects on RBCs.^[^ ^26^ ^]^ Therefore, as shown in this study, QE‐AgNPs are very hemocompatible due to the presence of QE as a capping agent on their surface, which protects the erythrocytes from oxidative damage^[^ ^27^ ^]^ and thus, can be a good candidate in the biomedical fields.

### 2.5 The Partial Thromboplastin Time (PTT) and Prothrombin Time (PT) Assays

**Figure 5:**
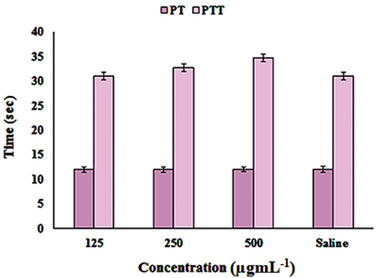
Anticoagulant activity of different concentrations of QE‐AgNPs solutions (125, 250, 500 µg mL^−1^) using PT and PTT assay in comparison to the controls (absence of QE‐AgNPs).

The interaction of the NPs with proteins of the coagulation cascade may alter the coagulation time and therefore evaluating the effect of nanoparticles on the coagulation pathways is very important. The PTT and PT assays are used to assess the impact of NPs on the intrinsic and extrinsic coagulation times, respectively.^[^ ^28^ ^]^ As shown in **Figure** **5**, the values of PTT or PT were expressed as mean ± SD (*n* = 3) and were compared to the controls (absence of NPs) by Tukey's test (analysis of variance (ANOVA)). Our results showed that the PT values for different concentrations of QE‐AgNPs were not significantly different from those of controls. Also, the concentrations 125 and 250 µg mL^−1^ did not change PTT significantly. These results recommend that the higher concentrations of QE‐AgNPs may impair the intrinsic pathways in the blood coagulation. Our obtained results were in accordance with the investigation of Martínez–Gutierrez on the effects of prepared AgNPs using gallic acid (24 nm) on coagulation pathways at a concentration of 67 µg mL^−1^, which they indicated that AgNPs at this concentration could not change PT or the extrinsic pathway, but PTT assay indicated inhibition of the intrinsic pathway of coagulation.^[^ ^29^ ^]^ However, in comparison to them, inhibition of the intrinsic pathway that slightly occurred by QE‐AgNPs was at the highest concentration of 500 µg mL^−1^, demonstrating good blood compatibility of QE‐AgNPs.

Therefore, for a better understanding of the role of surface functionalization and size of QE‐AgNPs on the initiation of coagulation factors, and the levels of stimulated coagulation factors, counting factors I (fibrinogen), III (tissue thromboplastin), V (Proaccelerin), VII (serum prothrombin conversion accelerator), VIII (antihemophilic factor (AHF)), IX (plasma thromboplastin component), XI (plasma thromboplastin antecedent), and XII (Hageman factor), more detailed studies are necessary. Especially, fibrinogen is a key protein in the coagulation cascade and interactions of nanoparticles with this protein may influence the coagulation cascade.^[^ ^28^ ^]^ Therefore, in the current study, we evaluated fibrinogen levels in treated blood samples with QE‐AgNPs. The obtained results showed a slight reduction of fibrinogen by 12 mg dL^−1^ compared to the control sample. Thus, according to the results, it can be said that the slight inhibition of the intrinsic pathway against QE‐AgNPs at the highest concentration of 500 µg mL^−1^ may be due to reduced fibrinogen levels.

### 2.6 The Anti‐Inflammatory Properties of QE‐AgNPs by Assay of Protein Denaturation (In Vitro)

**Figure 6:**
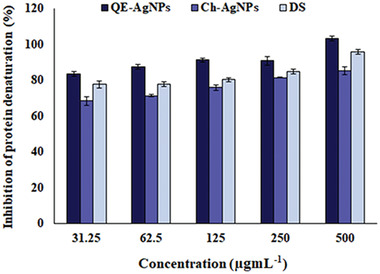
The in vitro anti‐inflammatory property of QE‐AgNPs by assay of inhibition of BSA protein denaturation at various concentrations 31.25, 62.5, 125, 250, and 500 µg mL^−1^ compared to DS and Ch‐AgNPs.

Denaturation of protein is a reason for created inflammation by different factors and diseases. This process can be inhibited using suitable inhibiting materials or anti‐inflammatory drugs.^[^ ^30^ ^]^ AgNPs are reported to have anti‐inflammatory activity and promote wound healing.^[^ ^31^, ^32^ ^]^ Therefore, in the current work, we were estimated the anti‐inflammatory effects of biogenic QE‐AgNPs compared to diclofenac sodium (DS) as anti‐inflammatory drugs by assaying inhibition of bovine serum albumin (BSA) protein denaturation in vitro. In this study, QE‐AgNPs showed 83.5% inhibition of BSA denaturation at the lowest concentration of 31.25 µg mL^−1^ compared to DS with 77.7% inhibition at a similar concentration, while, the maximum inhibition of BSA denaturation was obtained at a concentration of 500 µg mL^−1^ with 100% and 96% inhibition for QE‐AgNPs and DS, respectively (**Figure** **6**). At the same time, Ch‐AgNPs showed the maximum inhibition of BSA denaturation of 85.3% at concentration of 500 µg mL^−1^, which was lower than QE‐AgNPs and DS. Therefore, the higher anti‐inflammatory properties of QE‐AgNPs can be owing to the presence of QE as capping agents on the surface of these NPs. Our results are in line with previous studies that showed that quercetin is a potent anti‐inflammatory agent.^[^ ^27^, ^33^, ^34^, ^35^ ^]^ Therefore, QE‐AgNPs with strong anti‐inflammatory properties can be applied as a suitable anti‐inflammatory drug in biomedical applications.

## 3 Conclusion

In the current study, QE‐AgNPs have been biosynthesized using quercetin as a reducing and capping agent in the optimized conditions at 70 °C and pH 7. These synthesized QE‐AgNPs were characterized using UV‐vis, FTIR, XRD, and TEM techniques. QE‐AgNPs with spherical shape indicated the average particle size of 20 ± 3.6 nm. Furthermore, QE‐AgNPs revealed strong antioxidant, anti‐inflammatory, antimicrobial, and antifungal activity. QE‐AgNPs with anticoagulant activity was exhibited hemocompatibility by avoiding RBC lysis. Therefore, QE‐AgNPs with unique properties and high blood compatibility can be applied as a therapeutic agent for medical applications.

## 4 Experimental Section

### Materials

Silver nitrate (AgNO_3_), sodium hydroxide (NaOH), ethanol, hydrogen peroxide (H_2_O_2_), and aluminium chloride (AlCl_3_) were purchased from Merck. Quercetin, DPPH, Mueller Hinton agar and broth, Triton X100, sodium citrate, and BSA were obtained from Sigma‐Aldrich, USA. The chemically synthesized AgNPs (Ch‐AgNPs) with spherical shape and an average diameter of 8 nm were provided from the Iranian Nanomaterial Company (Mashhad).

### Synthesis of QE‐AgNPs

Silver nitrate (AgNO_3_) solution (1 × 10^−3^ m) was made in distilled water, and a stock solution of quercetin (1 × 10^−3^ m) was prepared in ethanol (50%). In order to synthesize the QE‐AgNPs, 1 mL of quercetin solution was mixed with 10 mL of AgNO_3_ solution, which was incubated at different pH (3, 5, 7, 10, and 12) and temperatures (37, 50, 70 °C). The alteration of the color of reaction solution from pale yellow to brown was considered as a sign of the QE‐AgNPs synthesis. pH and temperature and time of reaction were optimized.

### Characterization of QE‐AgNPs

The confirmation of synthesized QE‐AgNPs was recorded with UV‐vis spectroscopy (Shimadzu, Lambda UV mini‐1240 instrument) at a wavelength from 200 to 700 nm. The role of functional groups in the biosynthesis of QE‐AgNPs was determined by FTIR spectroscopy (IR Prestige‐21 instrument, Shimadzu Spectrometer, Kyoto, Japan), which was recognized at a resolution of 4 cm^−1^ and using KBr disk. The crystallinity of QE‐AgNPs was confirmed by the XRD spectrum (model: X'PertPro, Panalytical, Holland), which was operated with a current of 30 mA by Cu K^−1^ radiation at a voltage of 40 kV. TEM microscopy (Zeiss – EM10C, Germany, at a voltage of 80 kV) was applied for morphological analysis of QE‐AgNPs.

### Antioxidant Potential of QE‐AgNPs

The antioxidant potential of QE‐AgNPs was tested by scavenging of DPPH free radical and assay of QE content in vitro condition.^[^ ^36^ ^]^ Briefly, different concentrations of QE‐AgNPs, QE, and Ch‐AgNPs at 50, 100, 200, and 400 µg mL^−1^ were prepared in ethanol solution. Ascorbic acid (AA) was placed as a standard antioxidant compound. Then, 1 mL of each sample was mixed with 1 mL of 0.1 × 10^−3^ m DPPH radical solution (in ethanol). Then, these samples were incubated at room temperature for 30 min in dark condition. In the final step, a UV‐vis spectrophotometer at 517 nm (in reaction with DPPH) was applied to determine the absorption of samples against a blank. The mixed solutions of ethanol and DPPH radical were placed as control, and the percentage inhibition (%) of DPPH radical was assessed based on the following formula (Equation (1))

(1) $$\begin{array}{c} Inhibition\;\;of\;DPPH\;radical\;\left(\%\right)=\frac{\begin{array}{l} Absorbance\;\;of\;control\\ -\;Absorbance\;of\;sample\; \end{array}}{Absorbance\;of\;control}\;\times \;100 \end{array}$$

To determine the content of QE flavonoid in the biosynthesized QE‐AgNPs, 1 mL of AlCl_3_ solution (in ethanol) was mixed with 1 mL of QE‐AgNPs solution at the above concentrations. After 30 min of incubation, the absorbance of the samples was recorded at 415 nm. Finally, the QE content based on a standard curve of QE was determined as QE equivalent (µg QEE).

The hydrogen peroxide (H_2_O_2_) scavenging activity of QE‐AgNPs was examined according to the described method of Keshari and co‐workers.^[^ ^37^ ^]^ Briefly, 100 µL of QE‐AgNPs and Ch‐AgNPs solutions in phosphate buffer (50 × 10^−3^ m, pH 7.4) at different concentrations of 50, 100, 200, and 400 µg mL^−1^ were mixed with 300 µL phosphate buffer and 600 µL of H_2_O_2_ solution (2 × 10^−3^ m in phosphate buffer, 50 × 10^−3^ m, pH 7.4). Then, prepared samples were vortexed and incubated for 10 min at room temperature. After incubation time, the observance of samples was recorded at 230 nm and the percentage of H_2_O_2_ scavenging was estimated using Equation (1). AA was used as a standard compound.

### Antibacterial and Antifungal Properties of QE‐AgNPs

The antibacterial and antifungal properties of QE‐AgNPs were examined against several bacterial strains (*Pseudomonas aeruginosa* ATCC 27853, *Staphylococcus aureus* ATCC 29213, *Escherichia coli* ATCC 25922), and a fungus strain of *Candida albicans* using MIC and MBC methods by micro‐dilution assay (CLSI, 2017). Initially, 1.5 × 10^8^ CFU mL^−1^ of bacterial or fungal strains was cultured in each well (96‐well plates) and then was treated (as the serial dilution) with 400 µg mL^−1^ of QE‐AgNPs. After incubation (24 h) of the treated samples, bacterial growth was recorded. After 24 h of incubation, MBC tests were performed by culturing bacterial broth on the Mueller‐Hinton agar medium. Lack of bacterial growth under standardized conditions was placed as MBC.

### The In Vitro Hemolytic Activity Assay

The in vitro hemolytic assay of QE‐AgNPs and Ch‐AgNPS was done in accordance with Robert et al. method^[^ ^38^ ^]^ as follows; the fresh blood samples were prepared from 25–35 years old healthy adult volunteers by venipuncture and collected in the tubes contained K2EDTA (1.5 mg EDTA: 1 mL blood). The red blood cells (RBCs) suspension was attained by centrifugation of the blood samples at 800 g for 10 min and washed by normal saline. 200 µL of each concentration from NP solutions at different concentrations (62.5, 125, 250, 500, and 1000 µg mL^−1^) was added to 200 µL of 10% v/v RBCs suspensions and were incubated at 37 °C for 30 min. The treated samples after centrifugation (at 13 400 rpm for 5 min) were used for evaluation of the absorbance of free hemoglobin released from RBCs into the supernatant at 540 nm in a microplate reader. Triton X100 was used as a positive control with 100% RBCs lysis and normal saline as a negative control with no RBCs lysis. The values of hemolysis percentage were measured as follows (Equation (2))

(2) $$\begin{array}{c} Hemolytic\;activity\;\%\;=\frac{\left(\begin{array}{l} Absorbance\;of\;each\;concentration\;of\;\\ NPs\;-\;Absorbance\;of\;negative\;control \end{array}\right)}{\left(\begin{array}{l} Absorbance\;of\;positive\;control\;\\ -\;Absorbance\;of\;negative\;control \end{array}\right)}\;\times \;100 \end{array}$$

### The PTT and PT Assays

The contact of AgNPs with blood components like platelets altered platelet activation and caused thrombus generation.^[^ ^39^ ^]^ In the present study, the analysis of coagulation time was carried out to obtain some information about the impact of the synthesized QE‐AgNPs on blood coagulation. The anticoagulant activity of various concentrations of QE‐AgNPs suspension (125, 250, and 500 µg mL^−1^) was evaluated by using the PTT and PT tests with a little modification of the protocol described by Yang et al.. Briefly, the blood samples from healthy adult volunteers who did not get any medication at least 2 weeks, were taken by venipuncture and collected into sodium citrate‐containing tubes (9:1 v/v, blood: citrate). 100 µL of QE‐AgNPs were dispersed in 900 µL of whole blood and incubated for 30 min at 37 °C. Then, PTT, PT, and fibrinogen were measured by using the Coatron M2 Coagulometer (TECO, Germany). The results were expressed as mean ± SE with *n* = 3 and compared to the controls (absence of QE‐AgNPs).

### In Vitro Anti‐Inflammatory Activity by Protein Denaturation Assay

Inhibition of protein denaturation activity of QE‐AgNPs was tested by the described method of Vijayakumar et al. with minor modification.^[^ ^40^ ^]^ First, 1% BSA solution in Tris‐buffer (pH 6.5) and QE‐AgNPs’ aqueous suspension at various concentration of 31.25, 62.5, 125, 250, and 500 µg mL^−1^ were prepared. Then, 500 µL of QE‐AgNPs was mixed with 450 µL of BSA solution and incubated at 37 °C for 30 min, followed by 20 min heating at 70 °C. Finally prepared samples were cooled down, and absorbance was recorded at a wavelength of 660 nm. Distilled water was used as control. DS and urea were considered as positive and negative standard, respectively. The BSA denaturation's inhibition was calculated in percentage as follows (Equation (3))

(3) $$\begin{array}{c} Albumin\;denaturation\;inhibition\;\left(\%\right)\;=\frac{\left(\begin{array}{l} Absorbance\;of\;control\;\\ -\;Absorbance\;of\;the\;sample \end{array}\right)}{\left(Absorbance\;of\;control\right)}\;\times \;100 \end{array}$$

### Statistical Analysis

All results obtained from different analyses or tests were offered as mean ± standard error (SD), and one‐way ANOVA with Tukey's post‐test was used to analyze of data by using graph pad prism v. 5.0.4.533 (Graphpad Software, Inc). *p* Values less than 0.05 were considered as significant.

## Conflict of Interest

The authors declare no conflict of interest.

## Data Availability

Research data are not shared.
